# Longitudinal Predictors of Dental Caries, Periodontal Disease and Oral Health Quality of Life in People with HIV on ART: The OHART study

**DOI:** 10.21203/rs.3.rs-10060665/v1

**Published:** 2026-06-18

**Authors:** Temitope Tolulope Omolehinwa, Douglas E. Schaubel, Patricia M Corby, Sunday O Akintoye, Praise Okoh-Aihe, Afnan Khalifah, Marta Gabinskiy

**Affiliations:** University of Pennsylvania; University of Pennsylvania; University of Pittsburgh; University of Pennsylvania; University of Pennsylvania; King Abdulaziz University; University of Pennsylvania

**Keywords:** HIV, Antiretroviral therapy, dental caries, periodontal disease, salivary flow, oral health related quality of life, xerostomia related quality of life

## Abstract

**Background:**

Oral diseases are prevalent among people with HIV (PWH) receiving antiretroviral therapy (ART), yet longitudinal data on oral health outcomes and oral health-related quality of life (OHQoL) in this group are limited. This study investigates the longitudinal associations among demographic, clinical, and behavioral factors and oral health outcomes, as well as their impact on OHQoL in PWH on ART.

**Methods:**

In this two-year prospective observational study, 227 PWH on ART participated in five visits at 6-month intervals. We collected data on demographics; socioeconomic status; HIV history; ART; lifestyle risk factors such as tobacco, alcohol and recreational drug use; saliva flow rate; dental caries (DMFT and MT indices) and periodontal disease (clinical attach loss-CAL, Bleeding on probing-BOP and pocket depth-PD); OHQoL based on NHANES Oral Health Survey; and xerostomia-related quality of life. We used the generalized estimating equations version of linear regression to quantify associations among these factors.

**Results:**

DMFT (β = 1.05; p < 0.001) and MT (β = 0.89; p = 0.006) significantly increased with increasing age, among Blacks (DMFT: β = 3.25, p = 0.002; MT: β = 3.60, p = 0.003), and with increasing duration of HIV (p = 0.014). Higher salivary flow rate was associated with lower DMFT (p = 0.002) and MT (p < 0.001) scores, whereas bictegravir treatment was associated with increased DMFT (p = 0.046). Longer HIV duration correlated with decreased clinical attachment loss (p = 0.005), bleeding on probing (BOP; p = 0.036) and pocket depth (0.003). Bictegravir use was also correlated with decreased CAL (p = 0.019) and BOP% (p = 0.035). Hispanics showed reduced BOP (p = 0.010) when compared with non-Hispanics. Higher DMFT scores were associated with decreased OHQoL (p = 0.003).

**Conclusion:**

Adult PWH on ART have a high risk of dental caries and tooth loss, with hyposalivation/xerostomia contributing to lower OHQoL. While periodontal outcomes appeared favorable among individuals with longer HIV duration, aging, smoking, and specific ART exposures influenced oral health trajectories. These findings emphasize the importance of integrating oral health care into HIV treatment models and highlights the importance of addressing salivary dysfunction and caries risk in aging populations with HIV

## Introduction

Oral health is an essential component of overall health and well-being. Nevertheless, dental caries and periodontal disease remain major public health concerns in the United States and globally(1, 2), with substantial disparities across socioeconomic and vulnerable populations(3–6). Untreated dental caries is the most prevalent health condition worldwide, affecting approximately two billion people with permanent dentition(1, 7). Periodontitis is similarly widespread, affecting about one billion people globally(7).

People with HIV (PWH) represent a vulnerable population with multiple comorbidities(8, 9), despite significant improvements in survival and immune function with the widespread use of antiretroviral therapy (ART). This includes a high burden of oral diseases(10–12). PWH are believed to experience elevated rates of dental caries, hyposalivation or xerostomia, and possibly periodontal disease compared with individuals without HIV(11, 12).

Saliva plays a critical role in maintaining oral homeostasis through mechanical cleansing, buffering capacity, antimicrobial activity, and maintenance of oral pH(13). However, long-term ART use in PWH, has been associated with a decrease in saliva flow, with a subsequent potential increased risk of dental caries(13). Together, these oral conditions in PWH on ART can significantly impair oral health-related quality of life (OHQoL)(14, 15).

The landscape of periodontal disease in PWH has changed substantially in the ART era. While necrotizing gingivitis and periodontitis were very frequent in the pre ART era, chronic periodontal disease in PWH in the ART era is comparable to that of persons without HIV(16).

Existing evidence examining oral health outcomes in PWH is largely cross-sectional(15, 17, 18). These studies suggest that demographic factors (e.g., age, sex, income, education), behavioral factors (e.g., smoking, substance use), and HIV-related clinical indicators (e.g., CD4 count, viral load, ART duration) are associated with oral disease burden and affect OHQoL. However, longitudinal data evaluating how these factors interact over time in the ART era remain limited. Understanding the cumulative and dynamic relationships among demographic characteristics, salivary function, clinical indicators, and patient-reported outcomes, is essential to inform targeted prevention and management strategies.

The present study aimed to evaluate longitudinal associations between demographic characteristics, salivary flow rate, and clinical oral health indicators in PWH receiving ART, and to examine how these factors influence OHQoL over time.

## Methods

Study design and population: A prospective longitudinal observational study was conducted on n = 227 patients with HIV (PWH). Enrolled participants consented to be a part of the study, following approval from the Institutional Review Board of the University of Pennsylvania (IRB #: 843328), and were enrolled between February 2021 and January 2025. Participants were included if they had a confirmed diagnosis of HIV and had been on ART for at least 1 year. Participants were also included if they were at least 18 years of age and willing and able to consent to study and available for the study duration. Participants with orthodontic brackets on 8 or more teeth were excluded, due to difficulty with accurate periodontal probing with the orthodontic wires in.

Participants were seen every 6 months for 24 months for a total of 5 visits. Information obtained from participants included demographic (age, gender, ethnicity and race), and socioeconomic (education level, employment, insurance type, income, smoking history, recreational drug use and alcohol use) information. Clinical information obtained also included year of HIV diagnosis.

Stimulated saliva was collected every minute, for 5 minutes as previously described to assess saliva flow rate(19). Oral examination was performed by calibrated dental examiners and information obtained included clinical attachment loss, pocket depth and percentage bleeding on probing (BOP%) to assess periodontal health; and Decayed missing and filled teeth (DMFT) and Missing teeth (MT) indices to assess dental caries.

Oral Health- and xerostomia-related Quality of life (OHQoL and XeQoL respectively) were also assessed from participant responses on the NHANES Oral Health Survey 2012 (OHQoL) and a modified Xerostomia related QoL scale (XeQoL)(20, 21) (See supplemental data- Appendix 1 and 2).

De-identified participant data was input into a secure and monitored data capture platform, REDCap.

Statistical analysis: We summarized the study population using basic descriptive statistics, namely median and interquartile range (IQR) for continuous variables, and count and percentage for categorical factors. We modeled periodontal measures (mean clinical attachment loss (CAL); percentage bleeding-on-probing (BOP%); and mean pocket depth (PD) each as a function of the following predictors: demographics, baseline clinical variables, and lifestyle factors. This was accomplished using the generalized estimating equations (GEE) version of linear regression, which account for longitudinal measures (repeated within-patient over time). In addition, we modeled each of decayed-missing-and-filled teeth (DMFT) and missing teeth (MT) indices using the same modeling strategy. For descriptive purposes, we evaluated the pair-wise correlations among the afore-listed outcomes. Finally, we modeled each of the Oral Health Quality of Life (OHQoL) scores and the xerostomia related quality of life survey (XeQoL) score as outcomes. These models had the same set of predictors as the previously described periodontal and caries outcomes, but also included (in turn) the periodontal and caries metrics as predictors. For the OHQoL and XeQoL surveys, outcome variables were derived by averaging across a patient’s non-missing responses. Four questions from the Oral Health survey, which directly assessed OHQoL were analyzed: this includes OHQ.620, OHQ.640, OHQ.680 and OHQ.845. All regression models were adjusted for age, sex, race, ethnicity, calendar year of enrollment, duration of HIV, CD4 count, viral load, ART type (specifically bictegravir and dolutegravir), smoking, alcohol consumption and recreational drug use.

Statistical analysis was carried out in SAS (v9.4; Cary, NC).

## Results

**Figure 1.**
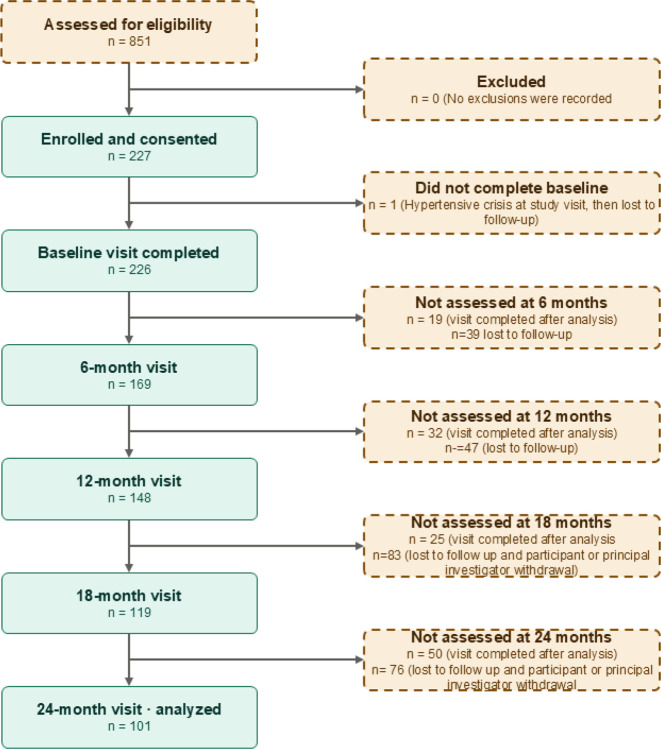
Participant flow and retention in the OHART cohort: Flow of people with HIV (PWH) receiving antiretroviral therapy (ART) through the 2-year study, from eligibility screening to the 24-month follow-up visit. Boxeson the left (Teal) indicate the number of participants completing each study visit based on data available at the time of analysis: 227 participants were enrolled, 226 completed the baseline visit, and 169,148, 119, and 101 completed the 6-, 12*. 18-, and 24-month visits, respectively. Retention at the 24-month visit at the time of analysis was 101/227 (44.5%). Boxeson right (Carton) indicate participants who had not yet completed the scheduled study visit at the time of analysis, were lost to follow-up, withdrew from the study, orwere withdrawn by the principal investigator. By study completion, an additional 50 participants had completed all five study visits, resulting in a final 24-month retention of 151/227 (66.5%).

Study population (Table 1): The study population consisted of n = 227 participants enrolled in the OHART study. At the time of analysis, 226 had completed the baseline study visit, while 169, 148, 119 and 101 participants had completed the 6-month, 12-, 18- and 24-month visits, respectively (Fig. 1). The median age of participants at enrollment was 57 years, with interquartile range (IQR) of (48, 63). The median number of years with HIV (at enrollment) was 23 years (IQR: 16, 29). Median CD4 + T-cell count for our participants across study visits was 638.0 cells/mm^3^ (IQR: 464.0, 853.5), with HIV RNA viral load averagely < 20 copies/mL (IQR: 0, < 20). Of the enrolled population, 75% were male; 63% were Black; and 8% were of Hispanic ethnicity. Approximately 49% of participants had a high school education, and 63% were unemployed at the time of enrollment. With respect to health insurance, 59% had Medicaid/Medicare, while 53% were covered under the Ryan White grant alone, or a combination of Ryan White and Medicare/Medicaid. Approximately 41% (40.5%) of participants were on bictegravir, and 16.3% on dolutegravir containing medications.

Predictors of periodontal outcomes (Table 2): Participants with longer duration of HIV had significantly decreased mean clinical attachment loss (CAL; p = 0.005), bleeding on probing percentage (BOP%; p = 0.036) and mean pocket depth (PD; p = 0.003). Older patients had significantly increased CAL (p < 0.001) and PD (p < 0.001) but not BOP% (p = 0.64). Hispanics experienced significantly decreased BOP% (p = 0.010) and near-significantly decreased PD (p = 0.095). BOP% increased significantly with each year of enrollment (p < 0.001). Smoking was associated with significantly increased CAL (p = 0.005) and near-significantly increased PD (p = 0.096). Neither of sex nor race were associated with CAL, BOP% or PD. Participants on bictegravir had significantly decreased CAL (p = 0.019) and BOP% (p = 0.035).

Predictors of dental caries and missing teeth (Table 3): Increased saliva flow rate was associated with a decreased decayed, missing and filled teeth score (DMFT; p = 0.002) as well as a significantly decreased number of missing teeth (MT; p < 0.001). Increased duration of HIV was associated with significantly increased DMFT (p = 0.014). Increased age at enrollment was associated with significantly increased DMFT (p < 0.001) and MT (p = 0.006). Black race was associated with significantly increased DMFT (p = 0.002) and MT (p = 0.003). Bictegravir use was associated with an increased DMFT (p = 0.046). Smoking was also associated significantly with increased MT (p = 0.019).

Correlation among dental and periodontal outcomes (Table 4): The three periodontal outcomes were all positively and significantly correlated, as would be expected, with the strongest correlation being between CAL and PD (ρ = 0.69; p < 0.001). DMFT was not significantly correlated with any of the periodontal measures, while MT was significantly correlated only with CAL (ρ = 0.14; p = 0.04). DMFT and MT had a very strong correlation (ρ = 0.82; p < 0.001), as expected.

Predictors of oral health quality-of-life (Table 5): Survey responses were scored such that higher oral health survey scores indicate better oral health quality of life (OHQoL). Increasing DMFT was associated with significantly decreased OHQoL (p = 0.003). Older patients tended to report greater OHQoL (p = 0.029). A higher number of MT was associated with a significant decrease in OHQoL (p = 0.011). BOP% was also associated with significantly decreased OHQoL (p = 0.004).

Predictors of xerostomia related quality of life (XeQoL) (Table 6): Responses to the XeQoL survey were scored such that higher scores reflect worse perception of XeQoL. Relative to other outcomes, there were fewer demonstrated associations with XeQoL. Alcohol use was associated with a near-significant increase in participants expression of worsening xerostomia symptoms (p = 0.079), while recreational drug use was associated with a significant decrease in XeQoL (p = 0.024). BOP% was also associated with worsening XeQoL.

## Discussion

This prospective observational longitudinal study characterized oral health trajectories among PWH on ART. In summary, longer duration of HIV was associated with a higher dental caries burden especially among Black participants, but lower levels of clinically assessed periodontal disease. Bictegravir use was associated with increased dental caries and decreased chronic periodontitis. Lower salivary flow was associated with increased risk of dental caries and more missing teeth. Older age was associated with increased dental caries and chronic periodontal disease burden, while smoking predicted increased clinical attachment loss and tooth loss. In addition, higher caries burden predicted poorer oral health-related quality of life. Interestingly, PWH who also used recreational drugs reported a better XeQoL.

Published cross-sectional studies from both small and large population studies, support our data, indicating a high caries burden among adults with HIV(17, 22, 23). For example, studies from Uganda, Rwanda, Portugal, Iran and the USA demonstrate caries prevalence rates of 50.5–83.7% in adult PWH(11, 24), and mean DMFT index scores up to 16.7(17, 24). Factors that contribute to increased caries risk in this population includes Black race and long duration of antiretroviral therapy (ART) use(17). Our data showed an association between dental caries and the two factors listed, however the data on association between duration of ART use and dental caries was not significant. Of note is that Black adults in the United States, including those without HIV, bear a significantly higher burden of untreated dental caries(25). Given this well-documented disparity, the observed association may reflect the combined influence of HIV-related factors and broader structural determinants of oral health, including access to care and socioeconomic disadvantage. Further, a previous study reported that those on ART > 5 years showed higher DMFT scores compared to those on treatment < 2 years(24). Integrase strand transfer inhibitors (INSTI) containing medications especially dolutegravir and bictegravir, which are recommended first line therapy for PWH, have especially been associated with dental caries(26, 27). Our findings suggest an association between INSTI-based regimens, particularly bictegravir-containing therapies, and increased caries risk. However, the underlying mechanisms remain unclear and should be investigated in future prospective mechanistic studies

In addition, most classes of ART have been associated with xerostomia or hyposalivation(27–29). Salivary hypofunction and xerostomia diminish the oral cavity’s buffering and antimicrobial capacity, facilitating the growth of acidogenic and cariogenic bacterial species and resulting in increased susceptibility to dental caries(30). Our study data demonstrated that dental caries correlated significantly with reduced salivary flow rate. This finding reaffirms the role of saliva as a critical defense mechanism in caries prevention, particularly for PWH taking ART.

We noted a trend of a possible association between a higher rate of dental caries and duration of ART use. We suspect that a strong association was not present due to relying on participant memory of when they commenced their individual ART medications, as we collected both history and current ART history, along with their start dates. For participants that did not recall their medication start date, we recorded it as having been on said medication for at least one year.

Greater caries burden was associated with poorer OHQoL, consistent with previous reports demonstrating the functional and psychosocial consequences of untreated dental disease. Dental caries and xerostomia can adversely affect eating, speaking, social interactions, and overall well-being. Interestingly, OHQoL was perceived as improved in older PWH. This may reflect adaptation to chronic symptoms, improved coping strategies, or greater engagement with dental care, among our older cohort.

Recreational drug use was unexpectedly associated with participants reporting better XeQoL. Because this finding lacks a clear biologic explanation and was based on self-reported outcomes, it should be interpreted cautiously and may reflect reporting differences or residual confounding.

Evidence regarding periodontal disease among PWH in the ART era remains mixed, with studies reporting both improvements and persistent disease despite viral suppression(31–34). In our cohort, longer HIV duration was associated with lower CAL, PD, and BOP, suggesting more favorable periodontal status. However, when BOP% was assessed cross- sectionally (per calendar year of enrollment), participants exhibited a greater tendency for bleeding on probing. These two time-related predictors should be interpreted separately.

One possible explanation is that longer HIV duration reflects sustained engagement in HIV care and prolonged exposure to effective ART, which may contribute to improved immune function and stabilization of periodontal tissues(34). Alternatively, a “placebo effect” of longitudinal studies, whereby enrolled participants adopt more vigorous oral hygiene practices following their initial visit as a result of study participation(35), may explain the discrepancy between calendar year of enrollment and the duration of HIV per year findings.

In addition, participants on bictegravir had significantly lower CAL and BOP. To the authors’ knowledge, there is currently no published literature identifying a specific role for bictegravir in the prevention of periodontal disease.

Age is a significant predictor of periodontal deterioration, with older participants demonstrating increased CAL and PD(33, 36, 37). The association between older age and greater CAL and PD is biologically plausible, reflecting cumulative exposure to periodontal risk factors and age-related changes in host immune and inflammatory responses(38). Smoking was also associated with periodontal deterioration, consistent with its established role as the strongest modifiable risk factor for periodontitis(39). Together, these findings highlight the importance of targeted periodontal surveillance and preventive care in an aging population of PWH.

Hispanic ethnicity was associated with lower gingival inflammation and a trend toward reduced periodontal pocketing. However, because Hispanic participants represented only a small proportion of the cohort (approximately 8%), these findings should be interpreted cautiously and require confirmation in larger and more diverse populations. The observed association may reflect unmeasured behavioral, environmental, or social factors that were not captured in the present study.

Of note is that unlike from findings in previous studies, our study did not find any significant association between HIV related clinical indicators including CD4 + T-cell count and HIV RNA (Viral load); and oral diseases.

Our findings suggest the need for a proactive, integrated approach to oral health in PWH, with a focus on preventive oral health care. This includes interprofessional collaboration between medical and dental professionals in coordinating care and ensuring referral of all PWH to dentists as soon as they commence ART. We also recommend implementing more frequent dental assessment in PWH, and encouraging the use of high fluoride toothpaste in this population in addition to topical fluoride treatment at dental visits. In addition, interventions aimed at preserving or stimulating salivary function including sialogogues, saliva substitutes, and fluoride therapies are crucial components of caries prevention in this population. Tobacco cessation should also be incorporated into the care of PWH as well as attention given to the oral care of older adults with HIV.

Another important clinical question to consider is if oral prophylaxis intervals for PWH be shortened to proactively address reversible gingival inflammation. We recommend considering more frequent dental cleanings every 3 months rather than standard biannual intervals for high-risk individuals with HIV.

### Limitations and Future Directions

While this study has many strengths including its prospective design, repeated oral health assessment, characterization of ART exposure and inclusion of both clinical and patient-reported oral health outcomes, as well as the longitudinal design strengthening our ability to infer temporal associations, the study is not without limitations. One of the major limitations on this study as previously reported was participant retention, therefore we have missing datapoints from study dropouts(19) (Fig. 1). Participant attrition resulted in missing longitudinal observations, which may have introduced selection bias despite the use of longitudinal analytic approaches. In addition, smoking, alcohol use, recreational drug use, and quality-of-life measures were self-reported and therefore susceptible to recall and social desirability bias. Information regarding ART duration and medication initiation dates was also not always available from medical records and occasionally relied on participant recall, potentially resulting in exposure misclassification. Finally, as an observational study, residual confounding cannot be excluded and causal relationships cannot be inferred.

## Conclusion

This prospective longitudinal observational study demonstrates that oral health outcomes among PWH receiving ART are influenced by a complex interplay of HIV duration, ART exposure, salivary function, aging, and behavioral factors. Reduced salivary flow and bictegravir use were associated with increased caries burden, whereas smoking and older age were important predictors of periodontal deterioration and tooth loss. Longer HIV duration was associated with more favorable periodontal measures. These findings support the need for integration of preventive oral healthcare into routine HIV management and highlight the need for targeted strategies to address caries risk, salivary dysfunction, and oral health-related quality of life in aging populations with HIV.

## Supplementary Material

Supplementary Files

This is a list of supplementary files associated with this preprint. Click to download.

- FinalSupplementaldata.docx

## Figures and Tables

**Table 1:** Baseline characteristics of study population (n = 227). Median and interquartile range (IQR) are shown for continuous variables; counts and percentages are shown for categorical variables

Variable	Units/category	Median or count	IQR or percent
Age	Years	57	(48, 63)
Duration of HIV (at time 0)	Years	23	(16, 29)
Sex	Female	57	25.1%
Race	Black	142	62.6%
	White	65	28.6%
	Asian	5	2.2%
	Multi	10	4.4%
	Other	5	2.2%
Ethnicity	Hispanic	18	8.0%
Education	Less than high school	24	10.6%
	High school	110	48.7%
	Associate's degree	37	16.4%
	Degree	55	24.3%
Employment	Unemployed	143	63.3%
	Part-time	54	23.9%
	Full-time	29	12.8%
Insurance type	Ryan White	120	53.1%
	Private	45	19.9%
	Medicare/Medicaid	134	59.3%
Income (annual)	< $20k	115	50.9%
	$20–35k	50	22.1%
	$35–50k	25	11.1%
	> $50k	36	15.9%
History of smoking	Smoker	86	38.1%
Alcohol use	Drinker	26	11.5%
Illicit drug use	Yes	76	33.6%
History of psychiatric disorders	Yes	86	37.9%
Hyperlipidemia	Yes	90	39.8%
Diabetes	Yes	46	20.4%
Hypertension	Yes	108	47.8%
CD4 count (time 0)	Cells per mm^3^	638.0	(464.0, 853.5)
Viral load (time 0)	Copies per mL	<20	(0, <20)
ART: dolutegravir	Yes	37	16.3%
ART: bictegravir	Yes	92	40.5%

**Table 2:** Longitudinal predictors of periodontal outcomes: Clinical attachment loss (CAL); percentage bleeding on probe (BOP%); and pocket depth (PD). Regression coefficients (three separate models for CAL, BOP% and PD) and corresponding p-values

Predictor	CAL		BOP%		PD	
	β	P	β	p	β	p
Duration of HIV (per year)	−0.02	0.005	−0.40	0.036	−0.01	0.003
Duration of ART (per year)	0.01	0.45	0.08	0.79	0.01	0.22
Calendar Year of enrollment (per year)	0.09	0.11	6.51	<0.001	−0.05	0.16
Age (per 5 years)	0.15	<0.001	−0.35	0.64	0.06	<0.001
Female (ref: male)	−0.15	0.54	3.06	0.58	−0.11	0.50
Hispanic (ref: not)	−0.03	0.93	−12.47	0.010	−0.19	0.095
Black (ref: White)	−0.17	0.25	3.93	0.28	0.06	0.59
Asian (ref: White)	0.20	0.52	12.03	0.24	0.23	0.40
Smoking (ref: no)	0.32	0.005	2.78	0.40	0.15	0.096
Alcohol (ref: none)	−0.06	0.81	−7.04	0.29	0.04	0.80
Recreational Drug use (vs none)	−0.20	0.057	−2.86	0.34	−0.12	0.12
ART: dolutegravir	−0.17	0.20	0.48	0.90	−0.10	0.24
ART: bictegravir	−0.29	0.019	−6.78	0.035	−0.13	0.14
CD4 count (per 100 cells per mm^3^)	0.01	0.43	−0.09	0.85	0.02	0.068
log (viral load + 1) (per unit)	0.02	0.61	0.11	0.90	0.02	0.28

**Table 3:** Longitudinal predictors of dental outcomes: Decayed, missing and filled teeth (DMFT) and Missing teeth (MT). Regression coefficients (two separate models for DMFT and MT) and corresponding p-values

		DMFT		MT	
Predictor	Scale or comparator	β	p	β	p
Saliva flow rate	per ml/min	−1.63	0.002	−2.14	<0.001
Duration of HIV	Per years	0.14	0.014	0.09	0.18
Duration of ART	Per year	0.06	0.53	−0.04	0.70
Calendar Year of enrollment	Per year	−0.57	0.28	0.58	0.34
Age	Per 5 years	1.05	<0.001	0.89	0.006
Female	vs male	1.25	0.42	2.53	0.17
Hispanic	Not Hispanic	−0.88	0.63	−1.94	0.22
Black	vs White	3.25	0.002	3.60	0.003
Asian	vs White	−0.87	0.71	−0.02	0.99
Smoking	vs Not	0.18	0.86	2.54	0.019
Alcohol use	vs Not	1.04	0.61	1.85	0.46
Recreational Drug use	vs Not	−0.91	0.34	−1.40	0.18
ART: dolutegravir	vs Not	0.89	0.39	0.10	0.93
ART: bictegravir	vs Not	2.07	0.046	1.82	0.14
CD4 count	per 100 cells per mm^3^	0.02	0.88	−0.23	0.17
log (viral load + 1)	Per unit	0.20	0.42	−0.01	0.97

**Table 4:** Correlation matrix relating periodontal and dental outcomes at baseline: Pairwise sample correlations (p values)

CAL	CAL	BOP%	PD	DMFT	MT
	1				
**BOP%**	0.40 (p<0.001)	1			
**PD**	0.69 (p<0.001)	0.21 (p = 0.003)	1		
**DMFT**	0.02 (p = 0.82)	−0.12 (p = 0.09)	−0.11 (p = 0.11)	1	
	
**MT**	0.14 (p = 0.04)	0.09 (p = 0.20)	−0.04 (p = 0.56)	0.82 (p<0.001)	1

**Table 5:** Longitudinal predictors of Oral Health-Related Quality of Life (OHQoL; higher scores indicate higher QoL): Regression coefficients and corresponding p-values.

		OHQoL	
Predictor	Scale or comparator	β	p
DMFT	Per unit	−0.02	0.003
Duration of HIV	Years	0.01	0.081
Duration of ART	Years	0.01	0.35
Calendar Year of enrollment	Year	−0.06	0.24
Age	Per 5 years	0.07	0.029
Female	vs male	−0.04	0.81
Hispanic	vs White	0.22	0.19
Black	vs White	−0.05	0.70
Asian	vs White	0.17	0.51
Smoking	vs Not	−0.13	0.29
Alcohol use	vs Not	−0.20	0.41
Recreational Drug use	vs Not	0.10	0.32
ART: dolutegravir	vs Not	−0.13	0.38
ART: bictegravir	vs Not	0.12	0.28
CD4 count	per 100 cells per mm^3^	−0.01	0.50
log (viral load + 1)	Per unit	0.02	0.55

When DMFT was replaced by missing teeth (MT) in the model, increasing MT was associated with asignifificant decrease in OHQoL (p = 0.011). When dental metrics were replaced by periodontalmeasures, only percent bleeding on probe had a signifificant (decreasing) effect on OHQoL (p = 0.004).

**Table 6:** Longitudinal predictors of Xerostomia score; higher scores indicate worse symptoms: Regression coefficients and corresponding p-values.

		Xerostomia related quality of life (XeQoL)	
Predictor	Scale or comparator	β	p
DMFT	Per unit	0.008	0.18
Duration of HIV	Years	−0.01	0.27
Duration of ART	Years	−0.01	0.43
Calendar Year of enrolment	Year	0.03	0.54
Age	Per 5 years	−0.03	0.22
Female	vs male	−0.06	0.64
Hispanic	vs White	−0.11	0.57
Black	vs White	−0.03	0.84
Asian	vs White	−0.02	0.96
Smoking	vs Not	0.08	0.46
Alcohol use	vs Not	0.34	0.079
Recreational Drug use	vs Not	−0.21	0.024
ART: dolutegravir	vs Not	0.03	0.83
ART: bictegravir	vs Not	−0.14	0.14
CD4 count	per 100 cells per mm^3^	0.01	0.41
log (viral load + 1)	Per unit	−0.05	0.13

When DMFT was replaced by missing teeth (MT) in the model, increasing MT was not associatedwith the XeQoL score (p = 0.13). When dental metrics were replaced by periodontal measures, onlypercent bleeding on probe had a signifificant (increasing) effect on the XeQoL score (p = 0.007).

## Data Availability

The datasets generated and/or analyzed during the current study are not publicly available due to participant privacy and confidentiality restrictions but are available from the corresponding author upon reasonable request and with appropriate institutional approvals. In accordance with NIH/NIDCR Data Management and Sharing Policy, de-identified data will be made available in a controlled-access research repository (or appropriate public repository, as applicable) at time of publication. The study is registered on ClinicalTrials.gov, where trial registration information and summary results will be available from October, 2026.

## List of Abbrevations

ART: Antiretroviral therapy
BOP%: Percentage of sites with bleeding on probing
CAL: Clinical attachment loss
DMFT: Decayed, missing and filled teeth
HIV: Human Immunodeficiency Virus
IRB: Institutional Review Board
IQR: Interquartile range
MT: Missing teeth
OHQoL: Oral health related quality of life
PD: Pocket Depth
PWH: People with HIV
XeQoL: Xerostomia related quality of life

## References

[1] Wu J, Chen J, Lv C, Zhou L. Global, regional, and National levels and trends in burden of dental caries and periodontal disease from 1990 to 2035: result from the global burden of disease study 2021. BMC Oral Health. 2025;25(1):844. 40442655 10.1186/s12903-025-06108-w PMC12123999

[2] Oral Health in America: Advances and Challenges. Bethesda (MD): National Institute of Dental and Craniofacial Research(US); 2021. 35020293

[3] Boyajyan V, Bilal U. Socioeconomic Disparities in Dental Caries Experience: The National Health and Nutrition Examination Survey 2011–2020. Am J Prev Med. 2025;70(5):108245. 41448289 10.1016/j.amepre.2025.108245

[4] Peres MA, Macpherson LMD, Weyant RJ, Daly B, Venturelli R, Mathur MR, Oral diseases: a global public health challenge. Lancet. 2019;394(10194):249–60. 31327369 10.1016/S0140-6736(19)31146-8

[5] Li Y, Zhang X, Wu Y, Song J. Association between social determinants of health and periodontitis: a population-based study. BMC Public Health. 2025;25(1):1398. 40229733 10.1186/s12889-025-22416-w PMC11998215

[6] Cianetti S, Valenti C, Orso M, Lomurno G, Nardone M, Lomurno AP, Systematic Review of the Literature on Dental Caries and Periodontal Disease in Socio-Economically Disadvantaged Individuals. Int J Environ Res Public Health. 2021;18(23). 10.3390/ijerph182312360 PMC8656978 34886085

[7] Trends in the global, regional, and national burden of oral conditions from 1990 to 2021: a systematic analysis for the Global Burden of Disease Study 2021. Lancet. 2025;405(10482):897–910. 40024264 10.1016/S0140-6736(24)02811-3

[8] Buysman EK, Kumar P, McNiff K, Goswami S, Paudel M, Prajapati G, Antiretroviral therapy among people with HIV with comorbidities in the United States: a retrospective cohort study. Curr Med Res Opin. 2023;39(11):1451–62. 37766585 10.1080/03007995.2023.2262379

[9] Gunter J, Callens S, De Wit S, Goffard JC, Moutschen M, Darcis G, Prevalence of non-infectious comorbidities in the HIV-positive population in Belgium: a multicenter, retrospective study. Acta Clin Belg. 2018;73(1):50–3. 28622754 10.1080/17843286.2017.1339965

[10] Coker MO, Cairo C, Garzino-Demo A. HIV-Associated Interactions Between Oral Microbiota and Mucosal Immune Cells: Knowledge Gaps and Future Directions. Front Immunol. 2021;12:676669. 34616391 10.3389/fimmu.2021.676669 PMC8488204

[11] Murererehe J, Malele-Kolis Y, Niragire F, Yengopal V. Prevalence of dental caries and associated risk factors among People Living with HIV/ AIDS and HIV uninfected adults at an HIV clinic in Kigali, Rwanda. PLoS ONE. 2023;18(4 April). 10.1371/journal.pone.0276245 PMC10079010 37023108

[12] Bartholo MF, Tenório JR, Andrade NS, Shibutani PP, Martins F, Gallottini M. Orofacial manifestations in Brazilian people living with HIV/AIDS under long-term antiretroviral therapy: a cross-sectional study. Oral Surg Oral Med Oral Pathol Oral Radiol. 2023;136(4):436–41. 37271609 10.1016/j.oooo.2023.05.001

[13] Gao X, Jiang S, Koh D, Hsu CY. Salivary biomarkers for dental caries. Periodontol 2000. 2016;70(1):128–41. 26662487 10.1111/prd.12100

[14] Murererehe J, Malele-Kolisa Y, Niragire F, Yengopal V. Oral health-related quality of life among people living with HIV and HIV-negative adults in Kigali, Rwanda: a comparative cross-sectional study. BMC Oral Health. 2024;24(1):128. 38273293 10.1186/s12903-023-03828-9 PMC10809602

[15] Rocha Trindade R, Marques J, Veiga M, Marques D, Mata A. HIV-1 impact on oral health-related quality of life: a cross-sectional study. AIDS Care. 2021;33(10):1321–8. 32715739 10.1080/09540121.2020.1798866

[16] Ryder MI, Shiboski C, Yao TJ, Moscicki AB. Current trends and new developments in HIV research and periodontal diseases. Periodontol 2000. 2020;82(1):65–77. 31850628 10.1111/prd.12321 PMC7441852

[17] Khodavirdizadeh Ghahremani S, Ghasemishayan R, Kia SJ, Khosousi Sani S, Khodavirdizadeh Ghahremani G. Correction: A cross-sectional study of oral health and disease prevalence in HIV-positive patients in Tabriz, Iran (2024). Sci Rep. 2025;15(1):34824. 41053366 10.1038/s41598-025-19521-6 PMC12501210

[18] Faé DS, de Aquino SN, Verner FS, Lemos CAA. Dental caries in HIV-infected children and adolescents: A systematic review with meta-analysis. Oral Dis. 2024;30(4):1756–64. 37357361 10.1111/odi.14637

[19] Omolehinwa TT, Akintoye SO, Gabinskiy M, Lo Re V, Mupparapu M, Urbina R, Oral health outcomes in an HIV cohort with comorbidities- implementation roadmap for a longitudinal prospective observational study. BMC Oral Health. 2023;23(1):763. 37848867 10.1186/s12903-023-03527-5 PMC10580527

[20] Lastrucci L, Bertocci S, Bini V, Borghesi S, De Majo R, Rampini A, Xerostomia Quality of Life Scale (XeQoLS) questionnaire: validation of Italian version in head and neck cancer patients. La Radiologia Medica. 2018;123(1):44–7. 28861706 10.1007/s11547-017-0798-7

[21] Henson BS, Inglehart MR, Eisbruch A, Ship JA. Preserved salivary output and xerostomia-related quality of life in head and neck cancer patients receiving parotid-sparing radiotherapy. Oral oncology. 2001;37(1):84–93. 11120488 10.1016/s1368-8375(00)00063-4

[22] Omolehinwa TT, Alamodi E, Akay-Espinoza C, Jordan-Sciutto KL, Adebiyi R, Stoopler ET, Cognitive impairment is associated with poorer oral health in people with HIV: evidence from a pilot study. Quintessence Int. 2026;57(2):140–50. 41211785 10.3290/j.qi.b6670849 PMC13449305

[23] Santo AE, Tagliaferro EP, Ambrosano GM, Meneghim MC, Pereira AC. Dental status of Portuguese HIV+ patients and related variables: a multivariate analysis. Oral Dis. 2010;16(2):176–84. 19744172 10.1111/j.1601-0825.2009.01622.x

[24] Kalanzi D, Mayanja-Kizza H, Nakanjako D, Mwesigwa CL, Ssenyonga R, Amaechi BT. Prevalence and factors associated with dental caries in patients attending an HIV care clinic in Uganda: A cross sectional study. BMC Oral Health. 2019;19(1). 10.1186/s12903-019-0847-9 PMC6642521 31324242

[25] Bashir NZ. Update on the prevalence of untreated caries in the US adult population, 2017–2020. J Am Dent Assoc. 2022;153(4):300–8. 34952680 10.1016/j.adaj.2021.09.004

[26] Gandhi RT, Landovitz RJ, Sax PE, Smith DM, Springer SA, Günthard HF, Antiretroviral Drugs for Treatment and Prevention of HIV in Adults: 2024 Recommendations of the International Antiviral Society-USA Panel. JAMA. 2025;333(7):609–28. 39616604 10.1001/jama.2024.24543

[27] Shiboski CH, Yao TJ, Russell JS, Ryder MI, Van Dyke RB, Seage GR, The association between oral disease and type of antiretroviral therapy among perinatally HIV-infected youth. AIDS. 2018;32(17):2497–505. 30096069 10.1097/QAD.0000000000001965 PMC6494108

[28] Navazesh M, Mulligan R, Karim R, Mack WJ, Ram S, Seirawan H, Effect of HAART on salivary gland function in the Women's Interagency HIV Study (WIHS). Copenhagen] :: Munksgaard; 2009. p. 52–60. 10.1111/j.1601-0825.2008.01456.x PMC2644059 19017280

[29] Diz Dios P, Scully C. Antiretroviral therapy: effects on orofacial health and health care.. Oral Diseases. 2014;20(2):136–45. 23530806 10.1111/odi.12093

[30] Meyle J, Dommisch H, Groeger S, Giacaman RA, Costalonga M, Herzberg M. The innate host response in caries and periodontitis. J Clin Periodontol. 2017;44(12):1215–25. 28727164 10.1111/jcpe.12781

[31] Gonçalves LS, de Carvalho Ferreira D, Vidal F, Souza RC, Gonçalves C, Pavan P, Correction to: Stage II and stage III periodontitis clinical burdens of HIV-1 undergoing antiretroviral therapy. Clin Oral Investig. 2022;26(5):4239. 10.1007/s00784-022-04410-3 PMC11837161 35257250

[32] Ceballos-Salobreña A, Gaitán-Cepeda LA, Ceballos-Garcia L, Lezama-Del Valle D. Oral lesions in HIV/AIDS patients undergoing highly active antiretroviral treatment including protease inhibitors: a new face of oral AIDS? AIDS Patient Care and STDs. 2000;14(12):627–35. 11119429 10.1089/10872910050206540

[33] de Almeida VL, Lima IFP, Ziegelmann PK, Paranhos LR, de Matos FR. Impact of highly active antiretroviral therapy on the prevalence of oral lesions in HIV-positive patients: a systematic review and meta-analysis. Int J Oral Maxillofac Surg. 2017;46(11):1497–504. 28684301 10.1016/j.ijom.2017.06.008

[34] Ntolou P, Pani P, Panis V, Madianos P, Vassilopoulos S. The effect of antiretroviral therapyon the periodontal conditions of patients with HIV infection: A systematic review and meta-analysis. J Clin Periodontol. 2023;50(2):170–82. 36261851 10.1111/jcpe.13735

[35] Preus HR, Al-Lami Q, Baelum V. Oral hygiene revisited. The clinical effect of a prolonged oral hygiene phase prior to periodontal therapy in periodontitis patients. A randomized clinical study. J Clin Periodontol. 2020;47(1):36–42. 31603245 10.1111/jcpe.13207

[36] Albandar JM. Disparities and social determinants of periodontal diseases. Periodontol 2000. 2025;98(1):125–37. 38217495 10.1111/prd.12547

[37] Sabine Elisabeth G, Yuxi Z, Jiawen Y, Lei W, Sabine R, Joerg M. Systemic, Lifestyle and Environmental Modifying Factors in the Pathogenesis of Periodontitis. J Periodontal Res. 2025. 10.1111/jre.70003 40511863

[38] Villoria GEM, Fischer RG, Tinoco EMB, Meyle J, Loos BG. Periodontal disease: A systemic condition. Periodontol 2000. 2024;96(1):7–19. 39494478 10.1111/prd.12616 PMC11579822

[39] Leite FRM, Nascimento GG, Scheutz F, López R. Effect of Smoking on Periodontitis: A Systematic Review and Meta-regression. American Journal of Preventive Medicine. 2018;54(6):831–41. 29656920 10.1016/j.amepre.2018.02.014

